# Growth and Cognitive Development in Tanzanian Children are Associated with Timing of Birth in Relation to Seasonal Malnutrition

**DOI:** 10.1016/j.jpeds.2024.114202

**Published:** 2024-12

**Authors:** Tarina Parpia, Sarah Elwood, Elizabeth T. Rogawski McQuade, Erling Svensen, Anne Wanjuhi, Samwel Jatosh, Eliwaza Bayo, Emanuel Hhando, Eric R. Houpt, Estomih Mduma, Mark D. DeBoer, Rebecca J. Scharf, James A. Platts-Mills

**Affiliations:** 1 Division of Infectious Diseases & International Health, University of Virginia, Charlottesville, VA; 2 Department of Epidemiology, Emory University, Atlanta, GA; 3 Haukeland University Hospital, Bergen, Norway; 4 Haydom Global Health Research Centre, Haydom, Tanzania; 5 Department of Pediatrics, University of Virginia, Charlottesville, VA

**Keywords:** cognitive development, food scarcity, malnutrition, global health, growth

## Abstract

**Objective:**

To evaluate in a rural Tanzanian birth cohort the association between birth timing in relation to the preharvest lean season and early-life growth and cognitive development.

**Study design:**

Children were enrolled within 14 days of birth and followed up for 18 months. Child anthropometry was measured every 3 months. The Malawi Developmental Assessment Test was administered at the end of follow-up. We estimated the association between timing of birth in the context of other early childhood risk factors and both growth and Malawi Developmental Assessment Test scores.

**Results:**

Children born in the preharvest months September and October had the lowest cognitive scores at 18 months, compared with birth in July and August (−1.05 change in overall Malawi Developmental Assessment Test development-for-age Z score, 95% CI: −1.23, −0.86). This association was observed for the language (−1.67 change in development-for-age Z score; 95% CI: −1.93, −1.40) and fine motor subcomponent scores (−1.67; 95% CI: −1.96, −1.38) but not for gross motor (−0.07; 95% CI: −0.23, 0.10) or social subcomponents (−0.07; 95% CI: −0.23, 0.10). Children born in September and October were the longest at birth but had the largest declines in growth Z scores during the first 6 months.

**Conclusions:**

There was a strong association between birth at the beginning of the preharvest season and poor growth and cognitive development. If these associations were mediated by the preharvest postnatal environment, targeted maternal and child interventions for children born during high-risk periods may improve these outcomes.

Trial registration: NCT03268902 (https://clinicaltrials.gov/study/NCT03268902).

Chronic malnutrition in childhood has been associated with long-term consequences including poor school performance and adult economic productivity, associations that are thought to be mediated by the impact of chronic malnutrition on cognitive development.^1^ Impaired linear growth is the most frequently used metric of chronic malnutrition and typically has been used as an outcome for interventional studies.^2^, ^3^, ^4^ There is also an established relationship between episodic acute malnutrition, typically measured by wasting, and stunting.^5^^,^^6^ Wasting is most common in the first 6 months of life and is higher in settings with seasonal rainfall, a predictor of seasonal variation in food availability.^7^ Birth timing in relation to seasonal rainfall is a risk factor for wasting, with peak wasting seen in children born before or during the rainy season.^7^ Birth season also has been associated with breast milk quality, immune development, and mortality into young adulthood.^8^, ^9^, ^10^ However, a direct association between birth timing in relation to seasonal malnutrition and cognitive development has not been described.

Communities with a single annual harvest are at particularly high risk for pre-harvest food insecurity and weight loss.^11^^,^^12^ Seasonal malnutrition has been associated with micronutrient and macronutrient deficits^12^ and long-term negative consequences on growth and educational attainment.^13^ A precise understanding of the relationship between seasonal malnutrition and child development could help inform seasonally-targeted interventions.^14^^,^^15^

Haydom, Tanzania, a rural, resource-poor setting with a maize-predominant diet and single annual harvest, was the site of the Early Life Interventions for Childhood Growth and Development in Tanzania trial.^16^, ^17^, ^18^ This randomized controlled trial enrolled more than 1000 children shortly after birth over a full calendar year to assess the effect of antimicrobial agents and micronutrient supplementation on length-for-age Z score at 18 months, with a cognitive assessment included as a secondary outcome. Enrollment started after a season of historically low rainfall and a poor harvest yield that led to substantial food insecurity. We have previously described risk factors for poor nutritional status at enrollment in this cohort.^17^ In the Haydom site, we have previously described seasonal food insecurity and associated malnutrition, with a rainy season from roughly November to April and harvest of beans in March and maize in May to July.^11^ In this analysis, we sought to evaluate the association between birth timing after a poor annual harvest, and postnatal cognitive development in the context of other risk factors and anthropometric trends to identify the value and optimal timing of seasonally targeted nutritional interventions.

## Methods

### Study Rationale and Design

We have previously described the rationale, design and protocol, and baseline characteristics of this trial.^16^^,^^17^ In brief, the study was a randomized, placebo-controlled, interventional trial to determine whether the receipt of (1) scheduled administration of antimicrobial agents (azithromycin and nitazoxanide) and/or (2) nicotinamide supplementation had an effect on linear growth at 18 months. The antimicrobials were administered together (vs corresponding placebos): azithromycin as a single 20 mg/kg dose at 6, 9, 12 and 15 months and nitazoxanide as a 3-day course of 100 mg twice daily at 12 and 15 months. The nicotinamide (vs corresponding placebo) was given to the mother daily as a 250-mg dose during nursing through child aged 6 months and then to the child daily as a 100-mg sachet through 18 months. Both the growth primary outcome and cognitive secondary outcome have been previously reported, with no benefit of either intervention seen.^18^^,^^19^ Mother/child dyads were enrolled within 14 days of the child's birth. At the initial visit, household information was collected including sociodemographic information, family assets, animal ownership, and water, sanitation, and hygiene practices. Standardized questionnaires were used monthly to characterize breast feeding practices, maternal and child illness, food insecurity, and health seeking behaviour. Food insecurity was defined as maternal report of worry during the prior month that the household would not have enough food.

The study protocol was approved by the National Institute for Medical Research or Tanzania and the Tanzanian Food and Drug Administration and the Institutional Review Board at the University of Virginia. Mothers gave written informed consent to participate either during pregnancy or at the time of enrollment.

### Anthropometry

Child anthropometry was measured at the initial visit and at 3, 6, 9, 12, 15, and 18 months of age. Weights were obtained using a digital scale. Lengths were measured with the child lying flat on a measuring board with 2 perpendicular boards, 1 fixed at the head and 1 adjustable at the feet. Two measurements were obtained, and if within 2 mm, the average of the 2 was used. If not within 2 mm, a third measurement was obtained, and the average of the 2 closest measurements was used. Head circumference was measured using a nondistensible tape above the eyebrows and ears and around the largest part of the back of the head. Finally, 5% of measurements were retaken by a supervisor to compare with the field team's measurements for quality assurance.^16^ For baseline characteristics with less than 5% missing data, mean values were single imputed to retain children in the adjusted analysis and retain comparability with the unadjusted analysis.^18^ Low birth weight was defined as a weight less than 2.5 kg at enrollment.

### Cognitive Testing

The Malawi Developmental Assessment Test (MDAT)^20^ was created to assess child development in children living in a rural sub-Saharan context, and we have previously described the adaptation, piloting, and validation of the test for use in Haydom.^19^ The MDAT includes direct observation and caregiver reports for tasks in 4 domains: gross motor, fine motor, language, and social. The assessments were performed in the child's primary language. Standardized MDAT Z-scores were calculated using version 1.1 of the MDAT Scoring Application.

### Statistical Analysis

Our sample for this analysis included all children with a valid length measurement and MDAT assessment at 18 months of age. First, in a planned analysis of risk factors for growth and cognitive outcomes in the Early Life Interventions for Childhood Growth and Development in Tanzania cohort, we estimated associations between baseline and early childhood characteristics and overall MDAT development-for-age Z scores (DAZ), MDAT subdomains, and length-for-age Z score (LAZ) at 18 months. For both outcomes, we used multivariable linear mixed-effects models, including in all models gender, tribe, ward of residence, birth order, preharvest or postharvest birth timing, a score that characterised socioeconomic status and included access to improved water and sanitation, household assets, maternal education, and income (WAMI),^21^ and a random effect for the study personnel performing the measurement or test. Based on rainfall and food availability, we defined the postharvest season as April through October and the preharvest season November through March. Because 15% of the 18-month assessments were delayed, we additionally adjusted for age at the time of the DAZ assessment,^19^ and in a sensitivity analysis, we additionally adjusted for enrollment LAZ. For the LAZ outcome, we additionally adjusted for enrollment LAZ and age at the time of the 18-month LAZ measurement. Next, in a post-hoc analysis, we fit the same models but respecifying birth timing with June/July as the referent, as these are the peak of the postharvest (ie, high food availability) months, and with the remainder of the calendar year divided into 2-month blocks. We examined effect modification by both WAMI quartile and maternal report of food insecurity. Finally, we used linear regression models to evaluate the association between birth timing and 6-month changes in anthropometry (birth to 6 months, 6 months to 12 months, and 12 months to 18 months), in each case adjusting for weight, length, and head circumference at the start of the interval. Finally, to understand the association between birth timing and static growth outcomes, we used unadjusted linear regression to evaluate the association between birth timing and the 18-month weight, length, and head circumference. Statistical analysis was performed using R, version 4.0.2.

## Results

**Figure 1:**
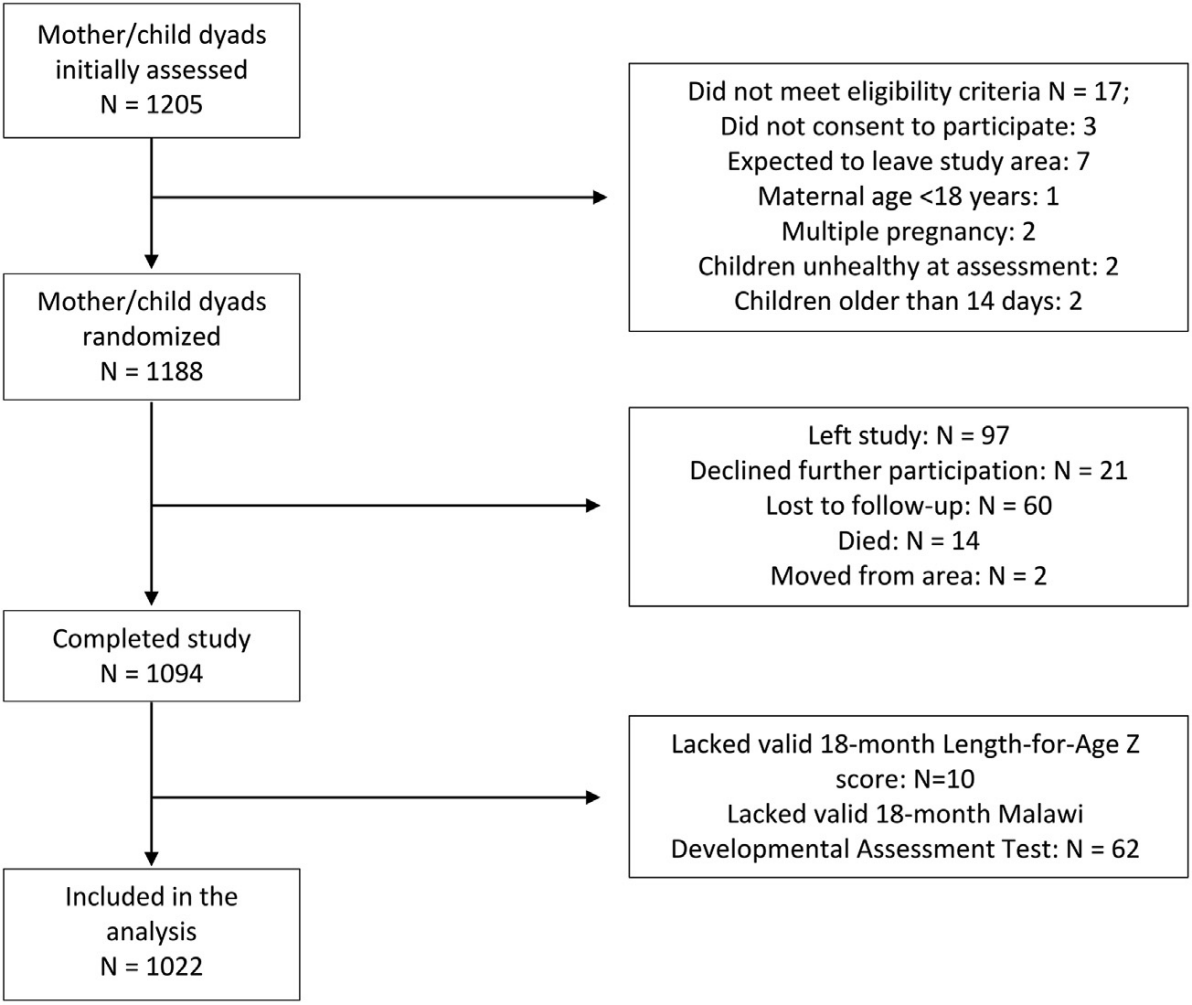
Children included in the analysis from the ELICIT cohort. *ELICIT*, Early Life Interventions for Childhood Growth and Development in Tanzania.

In total, 1188 children were enrolled from September 2017 to August 2018; of whom, 1022 (86.0%) were included in the analysis (Figure 1). Baseline sociodemographic characteristics were balanced across children born throughout the year of enrollment, and there was no clear association between birth timing and common childhood illnesses or hospitalizations (Table). The monthly proportion of mothers reporting any worry about having sufficient food at home was as low as 2.6% (27/1051) in July but increased to greater than 25% for November (32.8%; 38/116), December (37.1%; 63/170), January (32.4%; 101/312), and February (36.4%; 177/486). During the prior rainy season, 493.1 mm of rainfall was measured in Haydom, which was more than 2.5 standard deviations lower than the annual rainfall in the prior 6 seasons (mean 731.8 mm, SD: 93.0 mm).

**Table:** Baseline characteristics and early childhood risk factors in the ELICIT cohort by birth timing

	January-February 2018 (n = 281)	March-April 2018 (n = 217)	May-June 2018 (n = 146)	July-August 2018 (n = 129)	September-October 2017 (n = 83)	November-December 2017 (n = 166)
Baseline characteristics						
Female sex	136 (0.48)	113 (0.52)	85 (0.58)	65 (0.50)	47 (0.57)	85 (0.51)
Mother with ≥7 y of education	201 (0.72)	171 (0.79)	109 (0.75)	101 (0.78)	69 (0.83)	127 (0.77)
Firstborn child	33 (0.12)	34 (0.16)	29 (0.20)	27 (0.21)	17 (0.20)	33 (0.02)
Residence (ward)						
Endamilay	21 (0.25)	21 (0.25)	15 (0.18)	9 (0.11)	13 (0.16)	16 (0.19)
Garawja	27 (0.33)	25 (0.30)	19 (0.23)	12 (0.14)	1 (0.01)	16 (0.19)
Geterer	48 (0.58)	37 (0.45)	26 (0.31)	34 (0.41)	16 (0.19)	42 (0.51)
Hayderer	26 (0.31)	16 (0.19)	10 (0.12)	7 (0.08)	3 (0.04)	5 (0.06)
Haydom	61 (0.73)	35 (0.42)	17 (0.2)	26 (0.31)	29 (0.35)	33 (0.40)
Maghang	42 (0.51)	39 (0.47)	26 (0.31)	17 (0.20)	2 (0.02)	19 (0.23)
Mwanga	55 (0.66)	44 (0.53)	32 (0.39)	22 (0.27)	19 (0.23)	35 (0.42)
Other	1 (0.01)	NA	1 (0.01)	2 (0.02)	NA	NA
Datoga tribe affiliation	18 (0.06)	12 (0.06)	11 (0.08)	5 (0.04)	7 (0.08)	10 (0.06)
Age at 18-mo MDAT assessment (d)	559 (20)	546 (12)	544 (9)	550 (11)	556 (26)	610 (40)
WAMI score	0.29 (0.13)	0.30 (0.13)	0.31 (0.13)	0.33 (0.14)	0.33 (0.12)	0.31 (0.12)
Enrollment length-for-age Z score	−0.62 (0.91)	−0.60 (0.97)	−0.48 (0.97)	−0.32 (0.97)	−0.71 (0.95)	−0.79 (1.05)
Enrollment weight-for-age Z score	−0.71 (0.94)	−0.96 (1.05)	−0.85 (1.12)	−0.95 (0.88)	−0.42 (0.90)	−0.78 (1.10)
Enrollment head circumference-for-age Z score	−0.07 (0.95)	−0.17 (1.01)	0.04 (1.02)	0.01 (0.95)	0.17 (1.04)	0.05 (0.99)
Early childhood risk factors						
Duration of exclusive breastfeeding (mo) (median, IQR)	5 (4, 6)	5 (4, 6)	5 (4, 6)	6 (5, 7)	5 (3.5, 6.5)	5 (4, 6)
Food insecurity in first 12 mo of life∗	159 (56.6)	92 (42.4)	53 (36.3)	29 (22.5)	62 (74.7)	100 (60.2)
Diarrheal hospitalizations	26 (0.09)	17 (0.08)	11 (0.08)	11 (0.09)	10 (0.12)	11 (0.07)
Diarrheal hospitalizations in first 6 mo	22 (0.08)	14 (0.06)	10 (0.07)	8 (0.06)	8 (0.10)	10 (0.06)
Acute lower respiratory tract hospitalizations	35 (0.12)	20 (0.09)	12 (0.08)	14 (0.11)	9 (0.11)	21 (0.13)
Acute lower respiratory tract hospitalizations in first 6 mo	11 (0.04)	8 (0.04)	10 (0.07)	12 (0.09)	5 (0.06)	9 (0.05)
Any hospitalization	60 (0.21)	43 (0.20)	24 (0.16)	23 (0.18)	18 (0.22)	36 (0.22)

*ELICIT*, Early Life Interventions for Childhood Growth and Development in Tanzania; *MDAT*, Malawi Development Assessment Test.^21^

N (%) is shown for dichotomous variables and mean (SD) for continuous variables unless indicated.

∗ *P* < .001, Chi-squared test.

**Figure 2:**
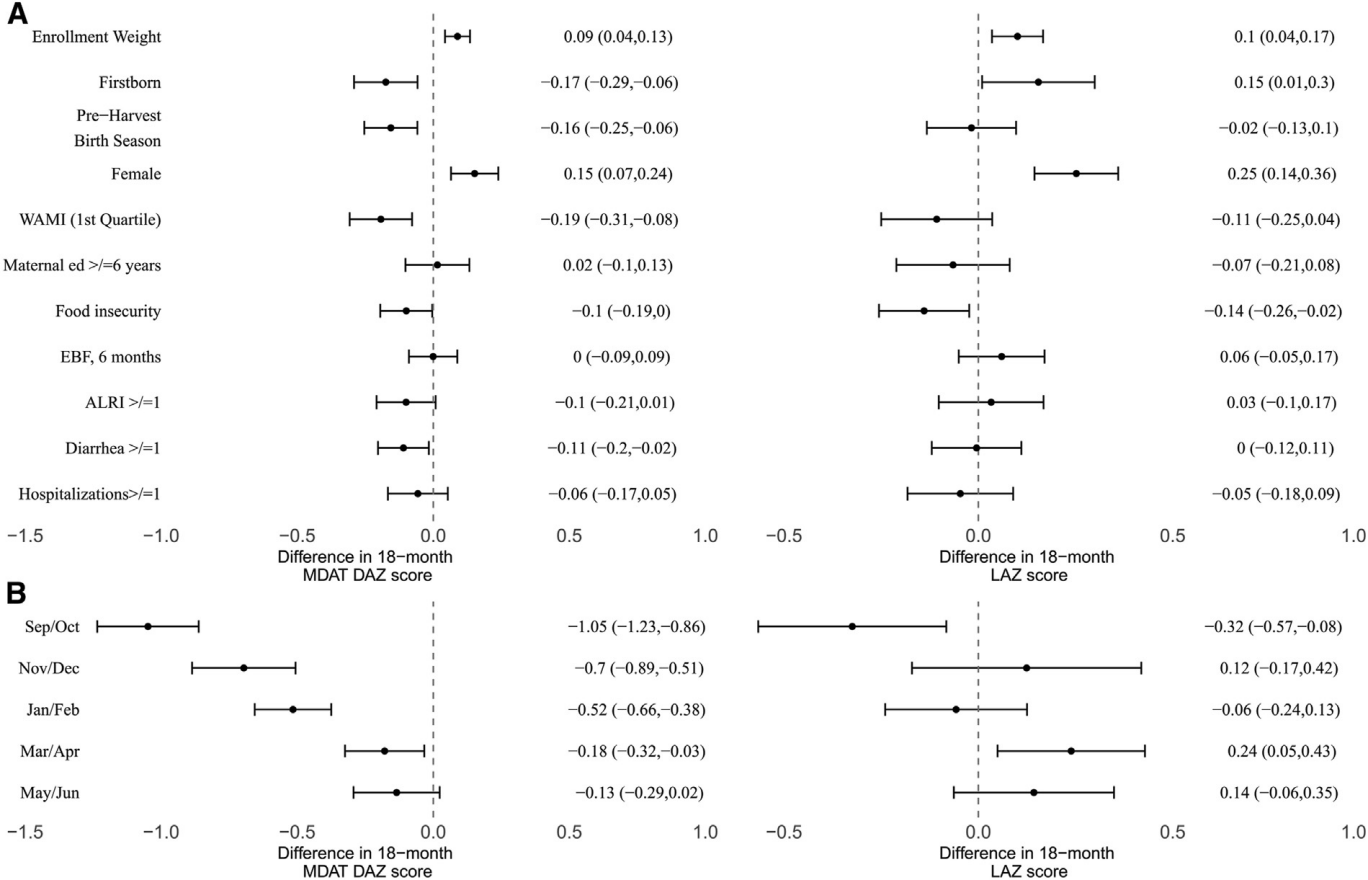
Association between baseline characteristics and early life risk factors (Panel **A**) and birth timing (Panel **B**) and both linear growth and cognitive outcomes in the ELICIT cohort. The preharvest birth season was defined as November through March.^21^ ALRI, acute lower respiratory tract infection; *EBF*, exclusive breastfeeding; *ELICIT*, Early Life Interventions for Childhood Growth and Development in Tanzania; *MDAT*, Malawi Development Assessment Test.

Higher enrollment weight was positively associated with both LAZ and overall MDAT DAZ at 18 months (Figure 2, A). Acute lower respiratory tract infections, hospitalizations, low birth weight, maternal education, and exclusive breastfeeding up to 6 months were not associated with overall DAZ score or LAZ at 18 months. A low WAMI score and a history of any prior diarrheal illness were associated with lower overall DAZ but had no association with LAZ at 18 months, while being firstborn was associated with a lower cognitive score at 18 months but a higher attained length at 18 months. Birth in the preharvest season was associated with a lower 18-month DAZ (−0.16; 95% CI: −0.25, −0.06 but not a lower 18-month LAZ (−0.02; 95% CI: −0.13, 0.10).

To evaluate better this association between birth season and these outcomes, we used 2-month cohorts (Figure 2, B). Birth in September/October 2017 was associated with the lowest DAZ at 18 months compared with the reference of July/August 2018 (−1.05 difference, 95% CI: −1.23, −0.86). This association was similar with or without adjustment for enrollment LAZ. Children born in each subsequent 2-month birth cohort after September/October had increasingly higher overall MDAT scores. This association between September/October birth and low DAZ scores was largest for children in the lowest WAMI quartile (−1.24 difference; 95% CI: −1.93, −0.56) but was seen across all quartiles. Similarly, the association between birth in September/October and the 18-month developmental score was similar between households with food insecurity (n = 495) (−0.98 difference; 95% CI: −1.27, −0.69) and households without food insecurity (n = 527) (−0.95 difference; −1.31, −0.58). The association was observed for the language (−1.67 difference, 95% CI: −1.93, −1.4) and fine motor (−1.67 difference, 95% CI: −1.96, −1.38) subcomponents but not for gross motor (−0.07 difference, 95% CI: −0.23, 0.1) and social (−0.07 difference, 95% CI: −0.23, 0.1).

**Figure 3:**
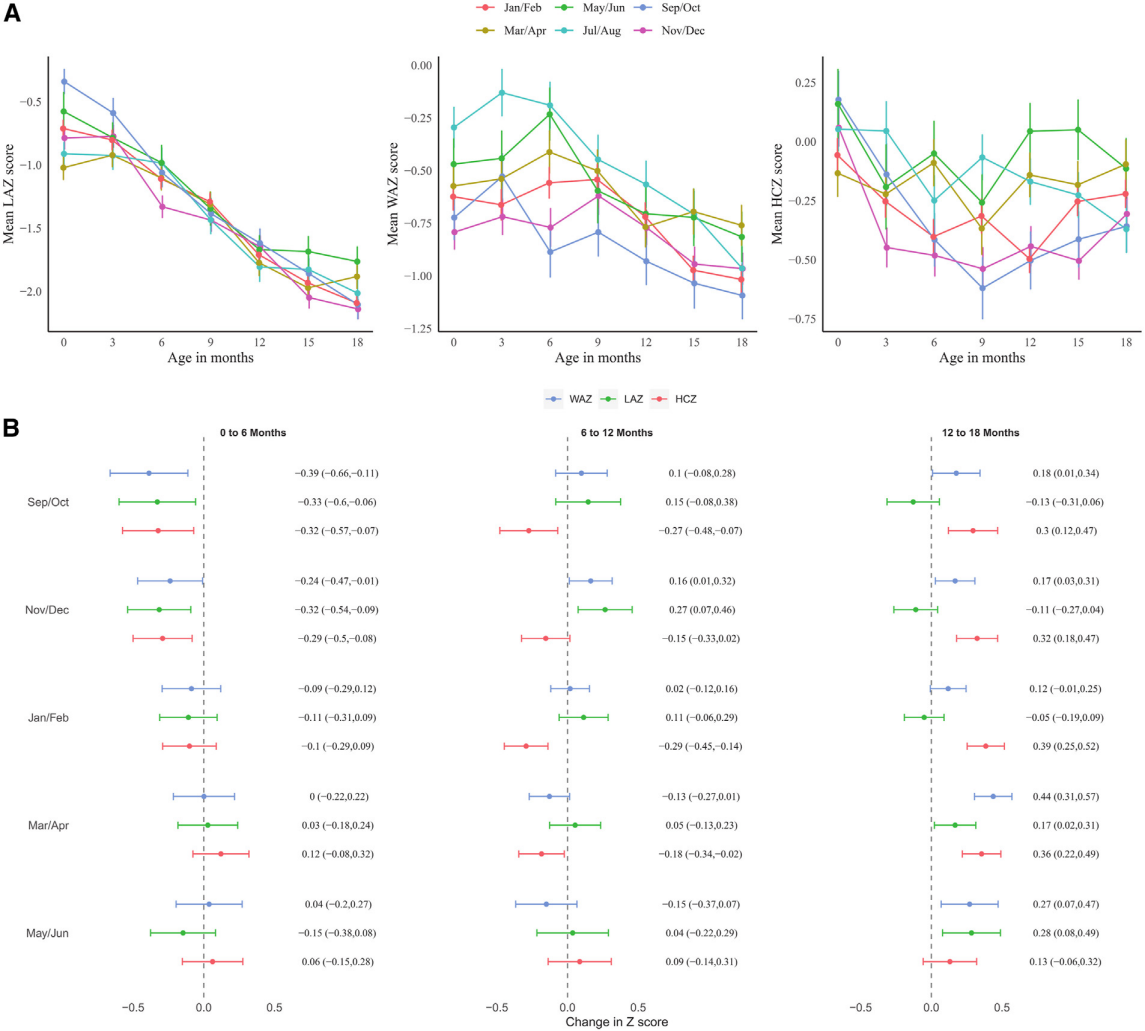
Anthropometric trajectories in the ELICIT cohort between birth and 18 months by 2-month birth season (Panel **A**) and association between birth season and 6-month anthropometry changes from birth to 18 months (Panel **B**). *ELICIT*, Early Life Interventions for Childhood Growth and Development in Tanzania; *HCZ*, head circumference-for-age Z score.

When adjusted for baseline LAZ, birth in September/October was associated with a lower 18-month LAZ score (−0.32 LAZ vs July/August; 95% CI: −0.57, −0.08) (Figure 2, B). Children born in September/October had the highest mean length and head circumference at enrollment but had the largest decline in weight, length, and head circumference Z scores from enrollment to 6 months of age, and the mean HCZ score for this cohort declined from the highest at birth to the lowest at 9 months of age (Figure 3). Enrolment WAZ as a continuous predictor (0.18 DAZ per one-unit increase; 95% CI: 0.13, 0.23) and change in WAZ from enrollment to 6 months of age (0.11 DAZ; 95% CI: 0.06, 0.16) were independently associated with 18-month MDAT DAZ. In unadjusted regressions (ie, without adjustment for baseline anthropometry), there were no associations between birth in September/October and 18-month length (−0.08 LAZ vs July/August; 95% CI: −0.35, 0.20), weight (−0.02 WAZ; 95% CI: −0.28, 0.25), or head circumference (−0.03 HCZ; 95% CI: −0.30, 0.24).

## Discussion

We identified a strong association between birth timing in relation to preharvest seasonal malnutrition and cognitive outcomes, with the lowest scores seen for children born in September and October, prior to seasonal rainfall and at the beginning of the preharvest lean season. This birth cohort was the tallest and had the largest head circumferences at enrollment but the lowest anthropometry trajectories in the first 6 months of life across all indices. Thus, the striking observed association between birth season and cognitive outcomes was most consistent with early postnatal malnutrition, and maternal and child nutritional interventions targeted to this time period for children born during high-risk periods of the year may have an outsized impact. In unadjusted analyses, there was no association between birth timing and attained weight, length, and head circumference at 18 months of age, suggesting that static anthropometric measures may miss important early differences in growth trajectories.

This study suggests that the association between significant food shortages and early-life cognitive outcomes is critically dependent on the timing of the exposure. Severe acute malnutrition has been associated with impaired cognitive development but is a distinct, severe clinical phenotype.^22^^,^^23^ As previously reported, children in this cohort born in September and October had the highest lengths at birth.^17^ This suggests that maternal nutritional status was good while these children were in utero, with third-trimester ponderal growth occurring during the period of highest food availability. However, these children spent their first 6 months of life during a period of food scarcity and insecurity, reflected by their poor length, weight, and head-circumference trajectories. This supports the suggestion that seasonal variation is an underappreciated driver of poor developmental outcomes in these settings.^15^ The timing of this effect was in contrast to a previously identified association between birth during the rainy season and mortality into young adulthood, which is thought to be an in-utero effect.^10^ There is evidence that physical growth in early infancy, as opposed to growth in the prenatal period or in late infancy, is associated with school-age IQ, supporting that this is a critical neurodevelopmental period.^24^

Because Early Life Interventions for Childhood Growth and Development in Tanzania included a nutritional intervention delivered via breastmilk, exclusive breastfeeding was emphasized to mothers by trained study personnel.^16^ As a result, the duration of exclusive breastfeeding was substantially longer than a prior observation cohort in the same community.^25^ It is possible that maternal malnutrition during the preharvest season and associated reduction breastmilk quantity or quality is one potential explanation for our findings.^26^ This suggests that seasonally-targeted maternal nutritional interventions in at-risk populations, in particular during the first 6 months of life or during exclusive breastfeeding, should be evaluated, with direct assessment of cognitive outcomes. Because seasonal wasting is observed in a wide range of settings, the relevance and public health impact of such a targeted intervention could be large.^7^ Additionally, better evidence is needed about the benefit of small-volume supplementation to children in the first 6 months of life in settings of severe malnutrition, where exclusive breastfeeding may be insufficient to meet nutritional demands.^27^

Neither socioeconomic status nor food insecurity modified the association between birth season and cognitive outcomes. The former suggests either that this association is related to factors apart from nutrition alone or that even households with relatively higher socioeconomic status still experience some scarcity of nutrition resources during the preharvest season given that the population in this area is uniformly impoverished. Food insecurity has been associated with lower breast milk intake in a similar setting in Western Kenya.^28^ In that study, a validated food insecurity score was used, whereas we used a single question that may not have been sufficient for accurate stratification of household access to food. Early childhood illness including diarrheal disease and respiratory infections can peak during the rainy season, but we did not see evidence that the September/October birth cohort had more childhood illnesses. Bacterial and protozoal enteric infections have been associated with poor growth,^29^^,^^30^ are often subclinical, including *Shigella*^31^ and *Cryptosporidium*,^32^ are often subclinical and peak during the rainy season, and it is possible that these infections influenced early child growth and development for these children more than those born at other times of the year.

While linear growth is not a direct estimate of child cognition, it has been negatively associated with cognitive performance of children^1^ and thus is frequently used as a convenient primary outcome in interventional studies targeting pathways associated with child growth and development. In this study, children born in September/October were the longest at birth. As a result, despite having the poorest linear growth during the study, these children had a similar mean attained height at 18 months to children born in July/August, despite the latter having the best cognitive outcomes. This suggests that attained height agnostic to the growth trajectory or starting point is an imperfect anthropometric marker for cognitive development.^14^ Additionally, we identified several other risk factors for poor cognitive development that were not associated with linear growth, including diarrheal illness. Thus, direct measurements of child cognition are important outcomes for interventional studies that hope to improve child development.^33^

This study benefited from a large birth cohort that was followed closely for growth and health outcomes and that received cognitive testing. Several limitations are important to note. First, the granular analysis of birth timing and growth and cognitive outcomes was a post-hoc analysis prompted by the findings of the broader risk factor assessment. However, the observation that birth in September/October was associated with both poor early growth trajectories and poor cognitive outcomes is compelling and could provide key preliminary data for future interventional studies. Because enrollment occurred during a single calendar year that followed a historically poor rainy season and harvest, it is possible that these findings may not be generalizable to other years in this setting, and that the observed effects could be driven by year-to-year factors in addition to seasonal ones. However, seasonal wasting is a broadly described phenomenon.^7^ Second, the study was suspended by Tanzanian regulatory agencies for 2 months, which delayed 18-month assessments for some children.^18^^,^^19^ We adjusted for the age of this assessment in all relevant analyses. It is also possible that 18-month developmental assessments were still influenced by seasonal variation, as children born in September and October would have experienced 2 preharvest seasons in the first 18 months of life, and there is evidence that MDAT scores and recover alongside recovery from wasting.^34^ Finally, we did not have markers of maternal malnutrition, which would have supported our hypothesis that this may be a driver of poor growth for children in the first 6 months of life during the period of highest food insecurity.

## Data Statement

Data sharing statement available at www.jpeds.com.

## CRediT authorship contribution statement

**Tarina Parpia:** Writing – original draft, Formal analysis, Data curation, Conceptualization. **Sarah Elwood:** Writing – review & editing, Formal analysis, Data curation. **Elizabeth T. Rogawski McQuade:** Writing – review & editing, Formal analysis, Data curation, Conceptualization. **Erling Svensen:** Writing – review & editing, Conceptualization. **Anne Wanjuhi:** Writing – review & editing, Investigation. **Samwel Jatosh:** Writing – review & editing, Investigation, Data curation. **Eliwaza Bayo:** Writing – review & editing, Investigation. **Emanuel Hhando:** Writing – review & editing, Investigation. **Eric R. Houpt:** Writing – review & editing, Conceptualization. **Estomih Mduma:** Writing – review & editing, Investigation, Funding acquisition, Conceptualization. **Mark D. DeBoer:** Writing – review & editing, Funding acquisition, Formal analysis, Conceptualization. **Rebecca J. Scharf:** Writing – review & editing, Formal analysis, Conceptualization. **James A. Platts-Mills:** Writing – original draft, Funding acquisition, Formal analysis, Conceptualization.

## Declaration of Competing Interest

This study was funded by the 10.13039/100000865Bill & Melinda Gates Foundation.

The authors declare no conflicts of interest.

## Supplementary Data

ELICIT observational STROBE_checklist_cohort

Data Statement
