# Supplementary material for: Growth and Cognitive Development in Tanzanian Children are Associated with Timing of Birth in Relation to Seasonal Malnutrition
Source: J Pediatr. 2024 Dec;275:114202. doi: 10.1016/j.jpeds.2024.114202 (PMC11582072; doi:10.1016/j.jpeds.2024.114202)
Supplement: Data Statement [file mmc2.docx]

Data Statement

Data required for the analysis reported in this manuscript will be made available upon request to other investigators on the site <https://clinepidb.org> as de-identified participant data at the time of manuscript publication.
